# From eHealth Technologies to Interventions

**DOI:** 10.2196/jmir.2050

**Published:** 2012-06-27

**Authors:** Rik Crutzen

**Affiliations:** ^1^Maastricht University/CAPHRIMaastrichtNetherlands

**Keywords:** eHealth technologies, interventions, Intervention Mapping

Van Gemert-Pijnen and colleagues recently presented an impressive viewpoint paper describing a holistic framework for the development of eHealth technologies, based on a review of existing eHealth frameworks [1]. As this review was limited to eHealth frameworks, the resulting holistic framework is focused on creating technologies instead of interventions. In my and others’ opinion, however, “interactive technologies are a tool, not a panacea” [2]. Therefore, it is useful – in *addition* to the holistic framework for the development of eHealth technologies – to look at intervention development approaches in general. Intervention Mapping, for example, focuses on the planning and development of effective interventions, not limited to eHealth technologies [3]. Comparable to the holistic framework, this is an iterative approach that takes into account social dynamics by incorporating stakeholders in a so-called linkage group. This linkage group consists of end users (e.g., patients) as well as intermediaries (e.g., nurse practitioners) and decision makers (e.g., department head, policy makers). It is striking to see the overlap between the activities mentioned in the holistic framework and the steps to be taken within Intervention Mapping (Table 1). Besides the overlap, Table 1 also indicates the differences in terminology used throughout the literature. Contextual inquiry, for example, entails “information gathering from the intended users and the environment in which the technology will be implemented” [1]. This is comparable to a key component of a needs assessment which encompasses “an effort to understand the character of the community, its members, and its strengths” [3]. During value specification, values are determined and ranked “based on the importance of finding solutions for the identified problems” [1]. One of the key tasks in the second step of Intervention Mapping is to select important and changeable determinants of behavioral and environmental outcomes [3].

More important than differences in terminology, however, are substantive differences between the holistic framework and Intervention Mapping. The two most important differences refer to the impact and uptake of eHealth technologies (both concepts mentioned in the title of the viewpoint paper [1]).

First, splitting up the design activity in two successive steps in Intervention Mapping is not merely a case of semantics or being more detailed. Active ingredients in interventions need to be based on theory-based intervention methods. These methods are to be translated into practical applications. Subsequently, these practical applications are incorporated while producing intervention programs. This results, finally, in intervention programs targeted at important and changeable determinants of behavioral and environmental outcomes, thereby increasing the likelihood of effective interventions (i.e., increasing their impact). Furthermore, it is insightful to look at active ingredients in interventions to unravel what works [4, 5], which can be facilitated by specifying methods and applications beforehand.

Second, with regard to uptake, the idea of Van Gemert-Pijnen and colleagues that long-term implementation requires business modeling is accepted with open arms. Although the role of cost-effectiveness analysis in health and medicine has been acknowledged for a while [6], business modeling is the next step that needs to be taken. Concepts and techniques from business modeling help to identify critical factors for the implementation, as Van Limburg and colleagues elaborated on in their companion paper [7].

Based on these differences, this letter should be seen as a call for integration: to treat interventions as technologies or even products that need to be marketed and require business modeling, as well as to focus on the content of interventions by means of producing intervention programs based on theory-based intervention methods that are translated into practical applications. In this way, eHealth interventions can effectively change health risk behaviors and their determinants [8] and their long-term public health potential can be actualized [9].

**Table 1:** Activities in the holistic framework and steps within Intervention Mapping

Holistic framework	Intervention Mapping
Contextual inquiry	Needs assessment
Value specification	Matrices of change objectives
Design	Theory-based intervention methods and practical applications
	Intervention program
Operationalization	Adoption and implementation
Summative evaluation	Evaluation
